# No Evidence of Sensory Neuropathy in a Traditional Mouse Model of Idiopathic Parkinson’s Disease

**DOI:** 10.3390/cells13100799

**Published:** 2024-05-08

**Authors:** Mahvish Faisal, Anna Rusetskaya, Liis Väli, Pille Taba, Ave Minajeva, Miriam A. Hickey

**Affiliations:** 1 Department of Pharmacology, Institute of Biomedicine and Translational Medicine, University of Tartu, 50411 Tartu, Estonia; mahvish.faisal@ut.ee; 2 Institute of Technology, University of Tartu, 50411 Tartu, Estonia; anna.rusetskaya@ut.ee; 3 Department of Neurology and Neurosurgery, Institute of Clinical Medicine, University of Tartu, 50406 Tartu, Estonia; liis.vali@ut.ee (L.V.); pille.taba@ut.ee (P.T.); 4 Estonia and Clinic of Neurology, Tartu University Hospital, 50406 Tartu, Estonia; 5 Department of Pathological Anatomy and Forensic Medicine, Institute of Biomedicine and Translational Medicine, Faculty of Medicine, University of Tartu, 50411 Tartu, Estonia; ave.minajeva@ut.ee

**Keywords:** heat sensation, cold sensation, touch sensation, sample size, repeated testing, unbiased stereology, motor coordination, parkinsonism, rotenone

## Abstract

Parkinson’s disease (PD) is the second-most common neurodegenerative disorder worldwide and is diagnosed based on motor impairments. Non-motor symptoms are also well-recognised in this disorder, and peripheral neuropathy is a frequent but poorly appreciated non-motor sign. Studying how central and peripheral sensory systems are affected can contribute to the development of targeted therapies and deepen our understanding of the pathophysiology of PD. Although the cause of sporadic PD is unknown, chronic exposure to the pesticide rotenone in humans increases the risk of developing the disease. Here, we aimed to investigate whether peripheral neuropathy is present in a traditional model of PD. Mice receiving intrastriatal rotenone showed greatly reduced dopamine terminals in the striatum and a reduction in tyrosine hydroxylase-positive neurons in the *Substantia nigra pars compacta* and developed progressive motor impairments in hindlimb stepping and rotarod but no change in spontaneous activity. Interestingly, repeated testing using gold-standard protocols showed no change in gut motility, a well-known non-motor symptom of PD. Importantly, we did not observe any change in heat, cold, or touch sensitivity, again based upon repeated testing with well-validated protocols that were statistically well powered. Therefore, this traditional model fails to replicate PD, and our data again reiterate the importance of the periphery to the disorder.

## 1. Introduction

Parkinson’s disease (PD) is a major neurodegenerative disease that shows a strong increase in risk with increasing age [1]. Traditionally, PD has been considered a motor disorder, and it is clinically diagnosed based on the presence of bradykinesia with at least one of the following: resting tremor, rigidity, or postural instability [1,2]. Motor dysfunction is largely due to dopaminergic neuronal loss in the *Substantia nigra pars compacta* (SNpc) [3]. However, extranigral changes in the central nervous system and also in the enteric and peripheral nervous systems likely contribute to the numerous non-motor symptoms [3]. A wide range of non-motor PD features, such as cognitive impairment, hallucinations, autonomic dysfunction, restless leg syndrome, disorders of sleep, and depression, are prevalent. Among these, the main non-motor symptoms frequently encountered include hyposmia and gastrointestinal (GI) dysfunction [4]. Impairments of peripheral sensory function also occur in PD and, indeed, disorders of the skin are more predictive of subsequent PD diagnosis than even olfactory deficits [5]. Moreover, aggregates of alpha-synuclein are observed in the skin and are similar to those observed in the brain [6], and the seeding capacity of PD-patient-skin-derived alpha-synuclein may be used for the antemortem diagnosis of PD and other synucleinopathies [7]. Critically, peripheral neuropathy may be considered unrelated to PD by patients and is, therefore, likely underdiagnosed [8], and indeed, treatments for non-motor symptoms in PD are a priority for development.

Peripheral neuropathy (PN) is a general term that indicates any dysfunction in the sensory, motor, and autonomic nerves of the peripheral nervous system [9]. PN can manifest as postural instability, loss of peripheral sensation, weakness, and/or pain, and it can be divided into small- (unmyelinated C fibres and thinly myelinated A δ fibres) and large-fibre neuropathy [10].

The cause of PD in the majority of patients is unknown, but several genetic risk factors have now been characterised, as well as several genes that cause rare autosomal dominant forms of PD [4]. Environmental factors, such as well-derived water, pesticides, herbicides, and farming activities, have also been reported as significant factors in causing PD [11]. Importantly, agricultural chemicals, such as rotenone and paraquat, which are pesticides and piscicides, have been shown to cause dopaminergic cell death in vivo and in vitro, and chronic exposure increases the risk of PD in humans [12,13].

Rotenone is a validated risk factor for PD [13]. It is a naturally occurring insecticide, pesticide, and piscicide extracted from the roots of plants of the genera *Lonchocarpus* and *Derris*. It is highly lipophilic and, therefore, easily crosses all biological membranes, including the blood–brain barrier [12]. It impairs oxidative phosphorylation by inhibiting mitochondrial electron transport chain complex I (ubiquinone oxidoreductase), leading to reduced ATP production and the formation of reactive oxygen species that can induce oxidative stress [14,15]. A major advantage of rotenone in modelling PD is that upon chronic ingestion of rotenone, alpha-synuclein accumulates in the enteric nervous system (ENS), which then induces transneuronal distribution of misfolded alpha-synuclein to the hind- (dorsal motor nucleus of the vagus) and midbrain (SNpc) of experimental animals [16,17,18]. Animal models exposed to rotenone and paraquat have received the most attention because chronic exposure increases the risk of PD in agricultural workers [13].

Several articles have been published where rotenone was administered intrastriatally (unilateral) to reproduce the neuropathological signs of PD, including dopaminergic cell loss in the SNpc [12,19,20,21,22,23]. This model has been shown to develop bilateral loss of tyrosine hydroxylase-positive neurons in SNpc and motor impairment [24]. Interestingly, this model suggested possible bidirectional communication between the gut and the brain, implying that it could induce other peripheral signs, including sensory neuropathy. Thus, here, we aimed to investigate whether this model also produced changes in sensation, which are increasingly recognised in PD patients. However, here, we report no changes in non-motor signs. Non-motor signs that were tested included whole-gut transition and sensitivity to heat, cold, and touch. Given that most preclinical trials of therapeutics use methyl-4-phenyl-1,2,3,6-tetrahydropyridine (MPTP) or 6-hydroxydopamine (6-OHDA) [25], which cause specific lesions of the SNpc, our data show that in mice, the degeneration of TH-positive neurons in SNpc alone is not sufficient to reproduce the full spectrum of PD. Importantly, we use gold-standard, statistically well-powered protocols that were tested repeatedly and for which we had calculated group sizes, in keeping with ARRIVE guidelines, to optimise our experimental design and increase the robustness of our results.

## 2. Materials and Methods

### 2.1. Animals

Male C57Bl/6N mice, initially aged 84 days and weighing 24–29 g (N = 22), were used in this study. The animals were housed at 21–23 °C under a 12 h light–dark cycle (7 am on and 7 pm off) with free access to *ad lib* food (V1534-300, ssniff Spezialdiäten GmbH, Soest, Germany) and water (reverse osmosis-treated and UV sterilised). The experimental procedures were carried out in accordance with the Estonian Animal Welfare Authorisation Committee (#198) according to the ARRIVE guidelines [26] and EU Directive 2010/63/EU.

### 2.2. Stereotaxic Surgery

Before stereotaxic surgery, the animals were divided into control and treated groups based on rotarod and grip strength performance during baseline training (see below). Two additional animals were used to confirm the injection site. For stereotaxic microinjections, the mice were anaesthetised using 2% isoflurane in oxygen and the skin overlying the skull was sterilised for surgery. Following placement in the stereotaxic frame, anaesthesia was maintained using approximately 1.5% isoflurane in oxygen. Ophthalmic ointment was placed on the eyes, and rectal temperature and respiration rate were monitored throughout. An incision was made in the skin overlying the skull and coordinates at the bregma and at AP + 2 mm and AP − 2 mm were obtained. The skull was then adjusted until the skull surface was flat (tolerance < 50 µm). The mice were treated with 5.4 µg of rotenone in 2 µL DMSO (N = 10) or vehicle only (DMSO N = 12) at the following coordinates: AP: +0.4 mm; ML: −2.5 mm and DV: −3.5 mm, which causes bilateral loss of TH-positive neurons in SNpc [24]. The solutions were protected carefully from light. A Hamilton syringe (Hamilton Americas & Pacific Rim, Hamilton Company Inc., Reno, NV, USA) was used to deliver rotenone or the vehicle over a period of 20 min (approximately 100 nL per minute). The needle was left in place for 2 min and then withdrawn over a further 2 min. Each mouse was administered with 0.5 mL saline (subcutaneous, sc) and carprofen (5 mg/kg sc) during the surgery to ensure appropriate hydration and analgesia, respectively, during recovery. The surgery procedure took approximately 1 h/mouse. Following suturing, mice were placed into a heated cage with food and water for approximately 1 h before returning them to their home cage. Analgesia was administered at regular intervals until 24 h after surgery.

### 2.3. General Health and Motor Testing

Mice were monitored daily and weighed every two days.

#### 2.3.1. Open Field

Spontaneous activity in a novel environment, over a period of 1 h, was analysed as per [27] at baseline and at 5, 10, and 15 weeks post-surgery.

#### 2.3.2. Grip Strength

Mice were placed onto the grip strength meter (TSE Systems, Berlin, Germany) and then encouraged to grip by pulling gently on the tail. Forelimb and all-four-limb grip strength was measured at baseline and 5, 10, and 15 weeks after surgery. Mice were given 5 trials, and the best three trials were used for analysis.

#### 2.3.3. Rotarod

An accelerating protocol was used, as per [28], as this is more sensitive to striatal impairment. All of the animals were pre-trained on the rotarod apparatus, comprising three trials per day over 4 consecutive days, with at least 20 min of rest between trials. Following surgery, rotarod performance was evaluated every two weeks (four trials over a period of 1 day, except in the case of week 2 where mice completed four trials per day over a period of 2 days).

#### 2.3.4. Hindlimb Stepping

Testing was carried out as per [29] at baseline and at 5, 10 and 15 weeks post-surgery. Importantly, levodopa administration improves performance in this task in unilateral 6-OHDA-lesioned mice [30], showing its superior sensitivity and specificity for models of PD.

#### 2.3.5. Gait Test

Footprinting was used to compare the gait of rotenone-treated mice with the control mice at the final time point (week 15) only. The hind- and forefeet of the mice were coated with green and blue non-toxic paints, respectively [31]. The animals were then allowed to walk along a 1 m long, 10 cm wide runway (with 10 cm high walls) into an enclosed chamber. All of the mice had training runs prior to the run used for analysis, although extra runs were used if footprints were indistinct. The footprint patterns were analysed for stride width, length, and overlap, as per [31].

### 2.4. Gut Motility

Colonic motility was examined by quantifying faecal pellet output over an 8 h period following gavage with carmine red [32], which is not absorbed from the lumen of the gut. Carmine red (300 µL per mouse; 6%; Sigma-Aldrich, Taufkirchen, Germany) was suspended in 0.5% methylcellulose (Sigma-Aldrich, Taufkirchen, Germany) and administered via oral gavage. Before administering, each animal was placed in an individual cage with some food and water. The number of regular and of red faecal pellets was counted at 60 min intervals for up to 8 h. GI motility was examined at baseline and at 5, 10, and 15 weeks after surgery.

### 2.5. Sensory Testing

The behavioural responses of rodents during hot-plate and cold-plate protocols are highly variable [33]. Thus, these protocols were not used. Importantly, for the Hargreaves, cold-plantar and von Frey tests, mice were habituated to their test environment for 1 h [33]. Mice that were grooming or sleeping were not tested—the personnel simply moved on to the next available mouse and returned at a later time during the session. For other tests, the mice were habituated to the testing room for 30 min.

#### 2.5.1. Hargreaves Test

The Hargreaves test (UGO Basile; intensity IR: 50) was carried out, as per review [33]. Each animal was placed gently into its own compartment with a glass floor and habituated for 1 h. The latency to remove the paw from the heat source over three trials was measured automatically at baseline and at 5, 10, and 15 weeks post-surgery.

#### 2.5.2. Tail Flick Test

The time taken for mice to flick their tail away from a heat source over two trials was measured automatically (Ugo Basile, Gemonio, Italy) at baseline and at 5, 10, and 15 weeks post-surgery. An IR heat intensity of 50 was used because preliminary testing revealed that lower intensities of 15, for example, resulted in latencies similar to “no intensity”.

#### 2.5.3. Cold Plantar Test

This test was conducted at weeks 10 and 15, and each hind paw was tested twice within each session. Gently, each animal was placed into its own compartment with a glass floor and habituated for 1 h [33,34]. The flat end of a dry-ice pellet was applied to the glass surface underneath the paw of the mouse and latency to withdraw was measured with a stopwatch (two timers controlled by two separate experimenters). Withdrawal was defined as any action to move the paw vertically or horizontally away from the cold site. An interval of at least 7 min was allowed between testing separate paws for a single mouse, and an interval of at least 15 min was allowed between trials on any single paw. The maximum time allowed for withdrawal was 20 s to avoid potential tissue damage.

#### 2.5.4. Hot- and Cold-Exploration Test

Empiric preference for heat or cold was assessed at weeks 10 and 15. The animals were allowed to freely explore an open arena (100 × 10 × 20 cm) over a period of 30 min, and their activity was recorded using Ethovision XT V8 (Noldus, Wageningen, The Netherlands). The arena comprised a glass floor, placed upon a hot plate at one end and ice packs at the other end. Preliminary experiments showed that floor temperatures ranged from 32 °C on one side to 10 °C on the other side and that temperatures remained constant over a period of 30 min. The proportion of time spent in each temperature zone was analysed automatically using Ethovision.

#### 2.5.5. Von Frey Test

In this test, each animal was placed gently into its own compartment and habituated for 1 h [33]. A monofilament was applied perpendicularly to the plantar surface of the hind paw until it buckled or the mouse responded. A response was considered positive if the animal exhibited any nocifensive behaviours, e.g., brisk paw withdrawal during application of the stimulus. The applied force began at 0.16 g, and testing followed the up–down method for the greatest sensitivity. Data were analysed using Up–Down Reader, which is an open-source program that efficiently determines 50% von Frey thresholds [35].

### 2.6. Pathological Analyses

The mice were euthanised at 16 weeks after surgery via cervical dislocation and decapitation. The brain was divided into hemispheres, and one hemisphere (ipsilateral to injection) was placed in fresh 4% paraformaldehyde. The duodenum and colon were also placed in fresh 4% paraformaldehyde. The samples were incubated at 4 °C, with rocking, for 48–72 h. The samples were then placed in 30% sucrose for a further 48–72 h and then briefly washed with 0.01 M PBS; the excess liquid was dried off, and they were then snap-frozen in liquid nitrogen. The samples were stored at minus 80 °C until processing. Serial coronal cryosections (40 μm) were taken, which were placed in cryoprotectant and stored at minus 20 °C until processing.

#### 2.6.1. Histochemistry

One section per mouse, approximately AP + 0.4, was stained for cresyl violet to confirm microinjection location, as per [27]. Photomicrographs were taken using a stereomicroscope (Zeiss Axio imager, Oberkochen, Germany) at 17× magnification to confirm appropriate needle placement.

#### 2.6.2. Immunohistochemistry and Stereology

In order to eliminate any bias, a section from the first six sections containing the SNpc was chosen at random (using the Excel Rand function). This section, and every 6th thereafter (thus, spaced at 240-micron distances), were used. The series of sections were washed, and then endogenous peroxidases were inactivated (1% H_2_O_2_ in 0.5% Triton X-100 in PBS; 20 min). The sections were then blocked using 5% goat serum (Jackson laboratories, Bar Harbor, ME, USA) in 0.5% TX-100 in 0.01 M PBS for 30 min and incubated in primary antibody overnight (anti-tyrosine hydroxylase (TH) antibody; Millipore Cat# AB152, RRID:AB_390204; 1:1000 in block, Burlington, MA, USA). On the following day, the sections were washed and incubated in secondary antibody (goat anti-rabbit biotinylated antibody; Jackson ImmunoResearch; 1:200 in blocking solution) for 2 h. Following washing, the sections were incubated in Vectastain Elite ABC Reagent in PBS containing 0.2% Triton X-100 for 2 h. The sections were washed and then developed in 0.03% 3-3-diaminobenzidine tetrahydrochloride containing 0.0006% H_2_O_2_ in 0.05 M Tris buffer, pH 7.6. Development was monitored carefully and then the sections were washed in TB to stop the reaction (approx. time to development: 16 min). The sections were mounted onto gelatin-coated slides, dehydrated and defatted and then cover-slipped. Control sections that were run in parallel and not exposed to primary antibodies showed no staining.

For stereology (3D estimation of SNpc TH-positive neurons), the SNpc was outlined in each of the sections using StereoInvestigator V5.00 on a Zeiss Z1 microscope at ×5 magnification. Briefly, the anterior-to-posterior extent of the SN was identified based on a standard mouse brain atlas (https://mouse.brain-map.org/static/atlas, accessed on 22 December 2022) using the following landmarks: rostral aspect of the SNpc began with the first TH-positive cells near the caudal end of the subthalamic nucleus; caudal SNpc ended where the retrorubral field became visible. The optical fractionator method (Stereoinvestigator V5.00) was used to count dopaminergic neurons. Cell counts were performed at 60× using a 1.4 NA lens and 1.4 NA oil condenser with a DVC real-time digital camera. Neurons were defined as having large, strongly TH-positive soma. The counting frames were distributed using a sampling grid of 150 × 150 µm. Counting frame sizes were 60 × 60 µm. Gunderson coefficients of error were always less than 0.1. Damage to two sections in one rotenone-treated mouse and the loss of a stereological series in one control-treated mouse resulted in group sizes of 9 and 11, respectively, for stereological data.

#### 2.6.3. Whole-Slide Scanning and 2D Estimation of SNpc TH-Positive Neurons

Slides were scanned using a 3DHistech Pannoramic Flash III 250 scanner (Budapest, Hungary) at a 20× magnification and analysed using SlideViewer (V2.7). The total number of TH-positive neurons in SNpc were counted per section, this total was multiplied by 6 (stereological series was 1:6), and these total numbers were added together to generate the mouse’ total number of SNpc neurons.

#### 2.6.4. Immunofluorescence

For GFAP (glial fibrillary acidic protein), two sections from the duodenum and colon were taken from each mouse and immunostained using anti-GFAP (1:500, Atlas Antibodies Cat# HPA056030, RRID:AB_2683015). Two sections from the brain (AP: −3.4 mm) were also stained in the same manner. These sections were adjacent to the sections used for stereology to enable the selection of SNpc and SNpr (*Substantia nigra pars reticulata*). Group sizes (N = 9 control, N = 9 rotenone) were smaller for pons GFAP as it was not present in some sections.

For LAMP1 (lysosomal-associated membrane protein 1), three sections from the colon were taken from each mouse and stained using anti-LAMP1 (1:1500, DSHB Antibodies Cat# 1D4B-c, RRID:AB_2134500).

For PGP9.5 (protein gene product 9.5), three sections from the colon were taken from each mouse and stained using anti-PGP9.5 (1:200, UCHL1 Antibodies Cat# aa171-220, RRID: AB_2210511).

For striatal TH, three sections from each mouse, approximately AP + 0.8 mm, were stained using anti-TH (Millipore Cat# AB152, RRID:AB_390204; 1:500 in block).

For immunofluorescence, staining protocols were as per [27]. Control sections that were not exposed to primary antibodies were always run in parallel, and no specific staining was observed in these sections. Counterstaining for immunofluorescence was performed using 1 µg/mL Hoechst-34580 dye (Sigma Aldrich) for 10 min, followed by washing in TB and then sections were mounted onto gelatin-coated slides.

### 2.7. Image Analyses for Immunofluorescence

For gut GFAP, Z-stacks through the depth of the area of interest were taken using an LSM780 confocal microscope (20×; 708.49 × 708.49 µm; 1.38 × 1.38 × 2.65 μm/pixel; frame size: 512 × 512 μm). Images were batch-processed in ImageJ version 1.53r. Briefly, channels were split, and the GFAP channel was made into a maximum-intensity projection, converted to 8-bit and then auto-thresholded. The longitudinal and circular muscle was outlined, and the percent area containing signal per image was quantified.

For colon LAMP1, Z-stacks through the depth of the area of interest were taken using an LSM780 confocal microscope (40×; 212.55 × 212.55 µm; 0.42 × 0.42 × 0.54 μm/pixel; frame size: 512 × 512 μm). The images were batch-processed in ImageJ, as above. The outer muscle layers (longitudinal and circular) were scored manually by a blinded individual for the extent of staining (0, +, ++).

For colon PGP9.5, photomicrographs of the colon were taken at 10× using cellSens Entry, V2.2 software (Olympus Life Science, Center Valley, PA, USA) on an Olympus IX70 microscope. To ensure consistency, all pictures were taken using the same settings, with calibrated brightness across the field of view and a black-balanced camera. For analysis, the images were converted to grayscale. The mean intensity of staining within outlined ROIs (regions of interest) of the longitudinal and circular muscle was quantified.

For brain GFAP, photomicrographs containing pons, SNpc, and SNpr were taken using an LSM780 confocal microscope (10×; 1024 × 1024 µm; 1.38 × 1.38 μm/pixel). The images were batch-processed in ImageJ; briefly, the channels were split and the GFAP channel made into maximum-intensity projections, converted to 8-bit, and then auto-thresholded. Based upon the ROI of SNpc from the adjacent TH-stained section used for stereology (see above), an ROI was drawn carefully around the SNpc and SNpr of GFAP-stained sections, and the percent area containing staining was quantified.

For the TH immunofluorescence of striatum, photomicrographs were taken at 4× using cellSens Entry, V2.2 software (Olympus Life Science, Center Valley, PA, USA) on an Olympus IX70 microscope. To ensure consistency, all of the pictures were taken using the same settings, with calibrated brightness across the field of view and a black-balanced camera. Photomicrographs of the sections were stitched together (Autostitch, accessed 1 November 2022, https://mattabrown.github.io/autostitch.html), and the integrated density of striatal fluorescence was analysed using ImageJ [36].

### 2.8. Statistics

All of the behavioural tests, pathological tests, and analyses were conducted by an experimenter blinded to the treatment. The null hypothesis that there was no significant difference between groups was rejected if *p* < 0.05. To compare one factor between two separate groups, unpaired *t*-tests were used, or Mann–Whitney U tests, if data were non-parametric. Where one factor was compared over time within a particular group, one-way ANOVAs were used, followed by appropriate post hoc testing. In the case of two factors, two-way ANOVAs followed by Šídák’s multiple comparisons tests were used. Three-way ANOVAs, followed by appropriate post hoc tests, were used where there were three factors (e.g., temperature zone, treatment, and time post microinjections for hot-and-cold exploration test). GraphPad Prism V9.3.1 was used for the majority of statistical analyses. Sample-size calculations for repeated tests were performed using GLIMMPSE software version 3.1.2 [37]. Sample-size calculations for single-timepoint testing were performed using ClinCalc (https://clincalc.com/, accessed on 31 May 2022) and G*Power version 3.1 [38].

In ClinCalc, the study group design was selected as “Two independent study groups”, the primary endpoint was continuous, alpha was set to 0.05 and power to 80%. For G*Power calculations, the test family was *t*-tests, which was set to independent and two-tailed, and power and sample sizes were calculated using an allocation ratio 1:1. Our trial adheres to ARRIVE 2.0 guidelines [26]. Data are available within File S1.

## 3. Results

### 3.1. Intrastriatal Rotenone Reduces Dopaminergic Terminals in Striatum and Loss of Dopaminergic Neurons in SNpc

**Figure 1:**
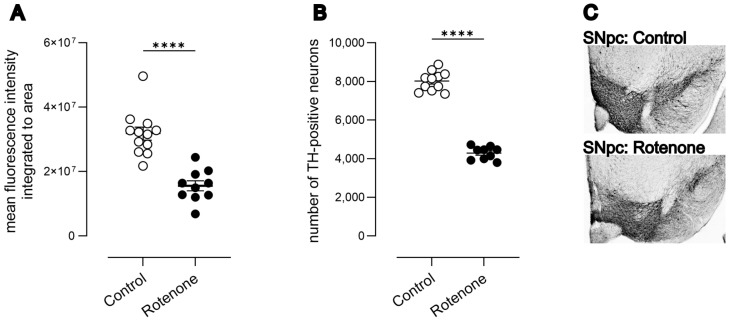
(**A**) Mean integrated fluorescence intensity of TH in striatae from control (N = 12) and rotenone-treated (N = 10) mice. Rotenone caused a significant reduction in striatal TH-positive terminals in striatum. **** *p* < 0.0001. Symbols are of individual mice, and horizontal bars show mean ± sem. (**B**) Rotenone-treated mice show a loss of dopaminergic (DAergic) neurons in SNpc (N = 9) compared with controls (N = 11). **** *p* < 0.0001. Symbols are of individual mice, and horizontal bars show mean ± sem. (**C**) Representative photomicrograph of DAergic neurons in the SNpc of a control mouse (top) and a representative photomicrograph showing loss of DAergic neurons in a rotenone-treated mouse (bottom).

Intrastriatal rotenone caused a significant reduction (49%) in striatal TH terminals ((Figure 1A) t (20) = 6.13, *p* < 0.0001).

Using unbiased 3D stereology based upon an optical fractionator, we quantified the number of TH-positive neurons in the SNpc. We observed a 50% reduction in TH-positive neurons of the SNpc ((Figure 1B) Mann–Whitney U = 0, N = 9–11; control median: 8153, 25–75% percentile: 7521–8387; rotenone median: 4451, 25–75% percentile: 3958–4560; *p* < 0.0001). The number of neurons in normal mice [19,24,39] and the reduction in TH-positive neurons in SNpc following intrastriatal rotenone is highly consistent with previously published data [19,24].

Due to the increasing prevalence of whole-slide-based quantifications, we also quantified TH-positive neurons based upon simple 2D counts from whole-slide scans. Using this latter method, we observed a lower number of TH-positive neurons in general and a 35% reduction in TH-positive neurons in rotenone-treated mice (see Supplementary Materials S1; Mann–Whitney U = 0, N = 9–11; control median: 2952, 25–75% percentile: 2844–3474; rotenone median: 1956, 25–75% percentile: 1830–2136; *p* < 0.0001). Thus, the relative difference between groups declined when using 2D counts, as shown previously [40]. Representative photomicrographs of SNpc from control- and rotenone-treated mice are shown in Figure 1C.

### 3.2. Intrastriatal Rotenone Causes Inflammation in the SN

**Figure 2:**
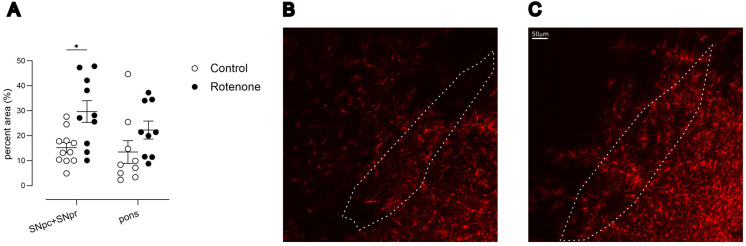
(**A**) GFAP fluorescence intensity, depicted as percent area above threshold, in SNpc + SNpr or in pons. Post hoc test: * *p* < 0.05. Symbols are of individual mice for SNpc (N = 11 control, N = 10 rotenone) or pons (N = 9 each). Horizontal bars show mean ± sem. SNpc, *Substantia nigra pars compacta*; SNpr, *Substantia nigra pars reticulata*. (**B**) Representative image of GFAP expression at 20×, in SN in a control-treated mouse. Outline shows SNpc, for reference, based upon an adjacent section stained for TH and used for stereology. (**C**) Representative image at 20×, showing an increase in GFAP expression in SN in a rotenone-treated mouse. Outline shows SNpc, for reference, based upon an adjacent section stained for TH and used for stereology. Scalebar in right = 50 μm, for both photomicrographs.

Inflammation in the SNpc is a consistent feature of PD, including the presence of reactive astrocytes [41]. This inflammation has long been considered a downstream response to the death of dopaminergic neurons [42]. We indeed noted a specific increase in GFAP-labelled astrocytes in the SNpc and SNpr of rotenone-treated mice compared with controls (treatment effect, F (1, 19) = 8.8, *p* < 0.01; Figure 2A) as we did not see any significant changes in GFAP expression in the more caudal pons.

### 3.3. Body Weight and Open Field Activity

**Figure 3:**
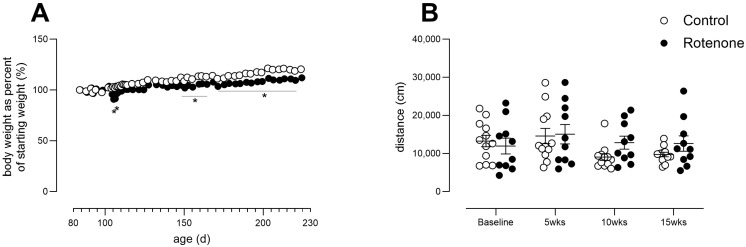
(**A**) Body weights from control- (N = 12) and rotenone-treated (N = 10) mice, expressed as percent of starting weight. Asterisks indicate significant differences between control and treated groups. Post hoc tests: * *p* < 0.05, ** *p* < 0.01. Symbols show group mean ± sem. Note that sem are obscured within symbols for many datapoints. (**B**) Activity in a novel environment (open field activity) over a period of 1 h in control- (N = 12) and rotenone-treated (N = 10) mice. No difference was detected between groups. Symbols are of individual mice, and horizontal bars show group mean ± sem.

After surgery, two rotenone-treated mice showed a reduction in weight, which was overcome by providing wet food for 2 days. Thereafter, rotenone-treated mice, as a group, gained less weight over time compared with DMSO-treated mice (Figure 3A; time X treatment interaction: F (50, 1000) = 11.14, *p* < 0.0001). With regard to spontaneous activity, no change was observed (Figure 3B, time x treatment F (3, 60) = 1.5, ns)

### 3.4. Intrastriatal Rotenone Induces Mild Changes in Gait and Does Not Affect Grip Strength

**Figure 4:**
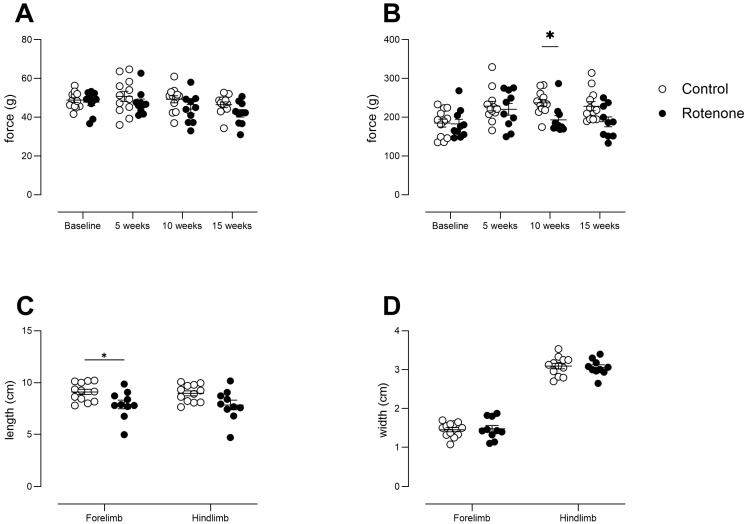
(**A**) Forepaw grip strength from control- (N = 12) and rotenone-treated (N = 10) mice. No difference between groups was observed. Symbols show individual mice, and horizontal bars show mean ± sem. (**B**) All-four-paw grip strength from control- (N = 12) and rotenone-treated (N = 10) mice. Although rotenone-treated mice, in general, showed reduced grip strength, it was only specifically significant at 10 weeks. * *p* < 0.05 Symbols show individual mice, and horizontal bars show mean ± sem. (**C**) Measurements of stride length of forelimbs and hindlimbs from control- (N = 12) and rotenone-treated (N = 10) mice. A minor deficit in forelimb stride length, only, was observed. * *p* < 0.05. Symbols show individual mice, and horizontal bars show mean ± sem. (**D**) Measurements of stride width of forelimbs and hindlimbs from control- (N = 12) and rotenone-treated (N = 10) mice. No differences between groups were observed. Symbols show individual mice, and horizontal bars show mean ± sem.

No consistent deficits in forelimb grip strength were observed in the rotenone-treated mice (Figure 4A time × treatment interaction, F (3, 60) = 0.76, ns). Although we note that all-four-paw grip strength tended to be lower in the rotenone-treated group (effect of treatment F (1, 20) = 5, *p* < 0.05), this did not reach significance when time was taken into account (Figure 4B time x treatment interaction F (3, 60) = 2.1, ns).

We noted a minor reduction in stride length in rotenone-treated mice, particularly forelimb stride length (Figure 4C; treatment effect, F (1, 20) = 5.8, *p* < 0.05); however, we did not see any change in hindlimb stride length, or in forelimb or hindlimb stride width (Figure 4C,D; stride width: treatment effect, F (1, 20) = 0.006, ns). Although unilateral and bilateral 6-OHDA mouse and rat models of PD have shown gait deficits [43], the deficits clearly correlate with the extent of dopamine loss in striatum, and it may be that, similar to open-field activity, gait analysis is not sufficiently sensitive to the deficits of our model. We also note that we used traditional paint-based analysis, which reduces the number of outcome measures. However, this *per se* is less likely to be the underlying cause of the lack of deficits, as others have used ink and shown deficits when the loss of TH-positive neurons in SNpc was profound [44].

### 3.5. Intrastriatal Rotenone Induces Progressive Motor Impairment in Sensitive Tests of Motor Coordination, Balance and Fine Movement

**Figure 5:**
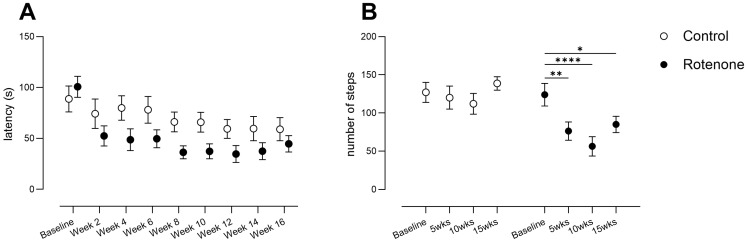
(**A**) Rotarod performance of control (N = 12) and rotenone-treated (N = 10) mice. Rotenone-treated mice showed a robust decline in performance. Symbols show group mean ± sem. (**B**) Number of continuous hindlimb steps in control- (N = 12) and rotenone-treated (N = 10) mice. A significant reduction in number of continuous hindlimb steps in rotenone-treated mice was observed. Asterisks indicate significant differences compared with baseline, * *p* < 0.05, ** *p* < 0.01, and **** *p* < 0.0001. Symbols show group mean ± sem.

Open-field activity was not sufficiently sensitive to detect motor impairment, and neither were gait analyses or grip strength measures. However, we also tested our mice with much more sensitive tasks that are more specific to striatal impairment. We found that the latency to fall from the accelerating rotarod decreased significantly in rotenone-treated mice (time x treatment interaction, F (8, 160) = 2.4, *p* < 0.02; Figure 5A); although this test suffered from high variability. Importantly, rotenone-treated mice showed a robust decrease in hindlimb stepping (time x treatment interaction: F (3, 60) = 3.2, *p* < 0.03; Figure 5B), a deficit also observed in genetic models of PD [45] and that is improved by levodopa in unilateral 6-OHDA-treated mice [30]. These deficits reiterate the importance of SNpc TH-positive neurons and striatal dopamine for fine motor coordination and balance. Indeed, multiple linear regression showed that of all motor outcome measures at endpoint (open field, forepaw grip strength, all-four-paw grip strength, hindlimb stepping, rotarod, hindlimb stride length, and hindlimb stride width), and with percent body weight at endpoint, only hindlimb stepping correlated with the number of dopaminergic SNpc neurons (F (1, 11) = 5.3, *p* < 0.05).

### 3.6. Intrastriatal Rotenone Causes No Change in Gastrointestinal Motility or Gastrointestinal Pathology

**Figure 6:**
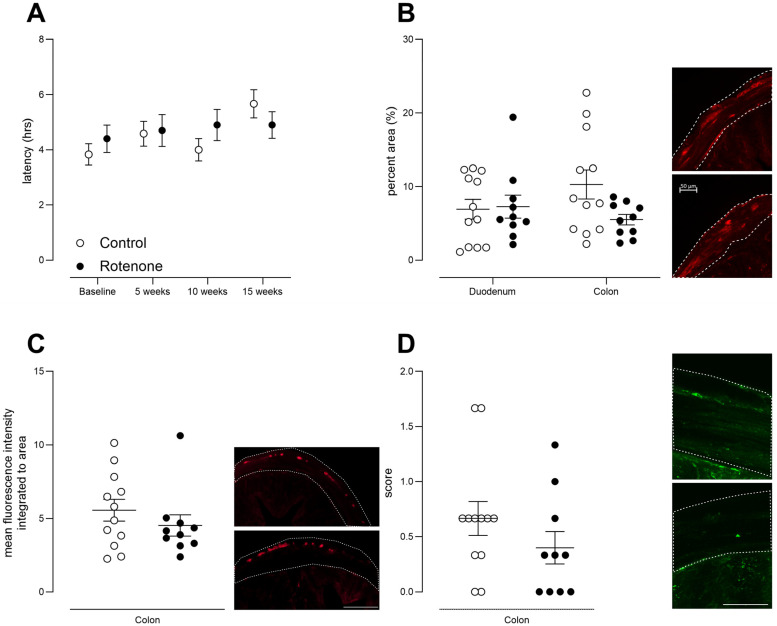
(**A**) Latency to expel red faecal pellets following administration of the non-absorbable dye carmine red in control- (N = 12) and rotenone-treated (N = 10) mice, i.e., whole-gut motility. No difference in latency was noted in repeatedly tested rotenone-treated mice compared with control-treated mice. Symbols are of group mean ± sem. (**B**) GFAP-positive staining, depicted as percent above background, in duodenum and colon from control- (N = 12) and rotenone-treated (N = 10) mice. No change was observed between groups. Symbols are of individual mice, and horizontal bars show group mean ± sem. Photomicrographs depict exemplar images from colon section, control (top) and rotenone (bottom, scalebar = 50 μm, for both photomicrographs). Dotted lines depict outer muscle layers (longitudinal + circular). (**C**) Mean integrated fluorescence intensity of PGP 9.5, a pan-neuronal marker that was used to detect enteric ganglia in longitudinal and circular muscle layers of colon from control (N = 12) and treated (N = 10) mice. No change was observed between groups. Symbols are of individual mice, and horizontal bars show group mean ± sem. Photomicrographs depict exemplar images from colon section, control (top) and rotenone (bottom; bottom, scalebar = 100 μm, for both photomicrographs). Dotted lines depict outer muscle layers (longitudinal + circular). (**D**) LAMP1-positive staining was scored on criteria of 0, + or ++ in outer longitudinal and circular muscle layers of colon from control- (N = 12) and rotenone-treated (N = 10) mice. No change was observed between groups. Symbols are of individual mice, and horizontal bars show group mean ± sem. Photomicrographs depict exemplar images from the colon, control (top) and rotenone (bottom, scale bar = 100 μm, for both photomicrographs). Dotted lines depict outer muscle layers (longitudinal + circular).

GI symptoms are relatively common in people with PD and can include constipation or a delay in gastric emptying [46,47]. In order to examine gut motility in our model, we administered a non-absorbable dye (carmine red) and then monitored faecal pellet output over a period of 8 h. This is a well-validated test that has been used in humans [48] and rodents [32,49]. No difference in latency to expel red faecal pellets was observed (Figure 6A, time x treatment F (3, 60) = 1.7, ns).

As GFAP expression is increased in PD patients in the gut [50], we further analysed GFAP expression in myenteric and intramuscular enteric glia in the duodenum and colon, which are thought to support myenteric neurons and modulate oxidative stress and inflammation [51]. In keeping with the lack of change in gut motility, we observed no change in GFAP expression in the duodenum or colon (Figure 6B, effect of treatment F (1, 20) = 1.8, ns; region x treatment F (1, 20) = 3.4, ns). We further confirmed a lack of effect of intrastriatal rotenone in the gut, as we found no change between groups in PGP9.5 staining (Figure 6C, t = 0.995, df = 20, ns), a pan-neuronal marker that reveals enteric ganglia, or in LAMP1, a traditional marker of lysosomes (Figure 6D, t = 1.24, df = 20, ns).

### 3.7. Intrastriatal Rotenone Induces No Change in Sensitivity to Heat or Cold

**Figure 7:**
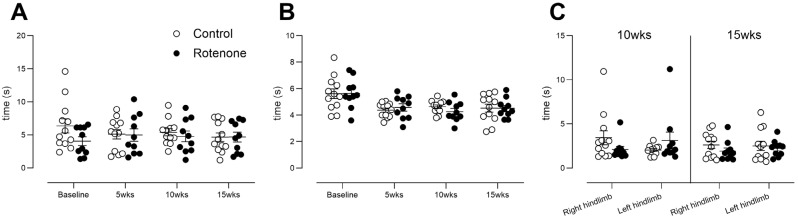
(**A**) The latency to withdraw hind paw from heat in control- (N = 12) and rotenone-treated (N = 10) mice. Symbols are of individual mice, and horizontal bars show group mean ± sem. (**B**) The latency to withdraw tail from heat in control- (N = 12) and rotenone-treated (N = 10) mice. Symbols represent individual mice, and horizontal bars show group mean ± sem. (**C**) The latency to withdraw hind paw from cold in control- (N = 12) and rotenone-treated (N = 10) mice. Although this model (intrastriatal rotenone) has been shown to result in bilateral SNpc TH-positive neuronal loss [24], we tested each hindlimb in the cold-plantar test, but this enhanced outcome also failed to reveal any deficit in sensation. Symbols represent individual mice, and horizontal bars show group mean ± sem.

The primary goal of this study was to assess peripheral sensation, behaviourally, in this traditional model of PD. Despite clear dopaminergic cell death and deficits in sensitive tests of fine motor movement, motor coordination, and balance, we did not observe any changes in heat sensation when using the Hargreaves test (Figure 7A; time x treatment interaction, F (3, 60) = 0.96, ns) or the tail flick (Figure 7B time x treatment interaction, F (3, 60) = 0.36, ns) test. We note that the Hargreaves test is thought to involve central processing but the tail flick test is more likely a spinal reflex [33]. Nonetheless, no deficits were observed in either outcome measure in the rotenone-treated group. Sensitivity to cold, tested similarly to the Hargreaves task, was also unchanged (Figure 7C; hindlimb x time x treatment interaction, F (1, 20) = 3.7, ns), despite increased cold pain sensitivity being a consistent finding in PD patients [52]. Thus, repeated testing over an extended period of time, using well-validated tests of heat and cold sensitivity, revealed no deficits in sensitivity to heat or cold in mice showing Parkinsonian motility impairments.

### 3.8. Intrastriatal Rotenone Causes No Change in Sensation of Touch

**Figure 8:**
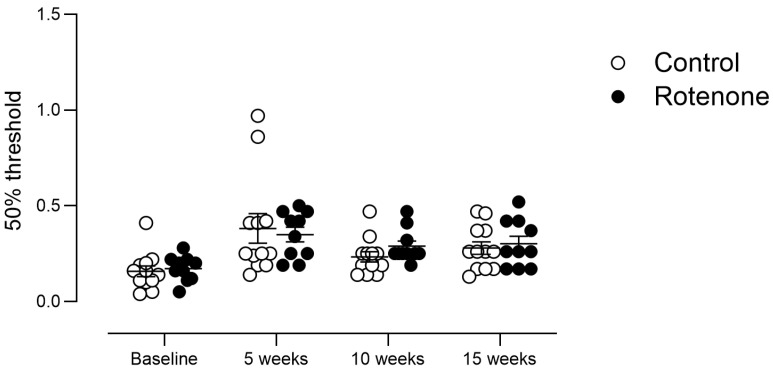
The 50% withdrawal threshold in control- (N = 12) and rotenone-treated (N = 10) mice. No change in sensation of punctate mechanical stimulus was noted in rotenone-treated mice compared with the controls. Symbols are of individual mice, and horizontal bars show group mean ± sem.

We then tested for the presence of mechanical allodynia and hyperalgesia using von Frey hairs applied manually to the plantar surface of the hind paw, which is the gold standard for testing mechanical threshold [33]. No significant difference between groups in touch sensation (Figure 8; time x treatment interaction, F (3, 60) = 0.4, ns) was observed, meaning that intrastriatal rotenone did not impact peripheral mechanical nociception.

### 3.9. No Empirical Preference for Hot or Cold

**Figure 9:**
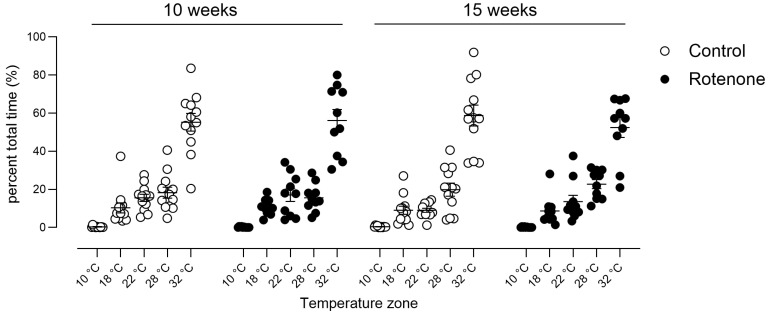
Spontaneous preference for hot or cold environments was measured in control- (N = 12) and rotenone-treated (N = 10) mice over a 30 min period. We did not detect any difference in preference for any particular temperature zone in rotenone-treated mice compared with controls. Symbols represent individual mice, and horizontal bars show group mean ± sem.

Double-mutant Pink1-/- SNCA^A53T^ mice showed a preference for lower temperatures compared with WT mice [53]; thus, we tested our mice for empiric preference for hot or cold environments. We did not see any difference in preference for heat or cold at either the 10- or 15-week time point in this novel test when we took time into account (Figure 9; temperature zone x treatment x time post-injection interaction, F (4, 80) = 1.3, ns) or when we analysed within each timepoint (10 weeks: temperature zone x treatment interaction F (4, 80) = 0.1, ns; 15 weeks: temperature zone x treatment interaction F (4, 80) = 0.7, ns).

### 3.10. Group Size Calculations

A separate preliminary experiment was conducted using a group of five mice to determine appropriate group sizes for each of our behavioural tests. These mice were tested once, and these data were then used to estimate group sizes that would be required to observe a rotenone-mediated change of 30% or 50%.

To then understand the effects of repeated testing, we used control data generated within this study (of the effects of intrastriatal rotenone) and again determined sample sizes that would be required to detect a change from the control of 30% or 50%, but this time based upon repeated testing using GLIMMPSE [37].

As shown in Table 1, repeated testing reduces group sizes required to see a 50% change from control for several tests (power set to 80% and alpha to 0.05). Moreover, for almost all of the tests that we used for our current experiments, we were well powered to detect treatment effects of 50%. Finally, our calculations suggest that in order to detect a 50% change from control, group sizes of 7–10 are sufficient, whereas group sizes of 16–25 are required to detect a change of 30%, with 80% power.

Thus, we have tested large groups of mice using robust, gold-standard behavioural protocols that are well-validated for mice. This testing generated highly statistically robust data that failed to reveal any behavioural sensory deficits in mice that nevertheless display Parkinsonian motor disorders.

## 4. Discussion

PD has historically been characterised as a neurodegenerative disorder that primarily affects the brain [1,3,4,46]. Given that central models of PD are primarily used for assessment of therapeutics in PD [25], and that previous data suggested non-motor deficits following central administration of rotenone [24], in this study, we sought to comprehensively analyse sensory behaviour following the intrastriatal administration of rotenone.

Several of the motor symptoms of PD are caused by the loss of DA-containing neurons projecting from the *Substantia nigra* to the dorsal striatum, and patients typically have almost 70% TH-positive neuronal loss in SNpc [54]. Thus, most animal models where a neurotoxin induces a strong and rapid cell loss in the SNpc show motor symptoms [12]. Here, all of the animals injected with rotenone were effectively lesioned, as they showed a 50% loss in DA neurons in the SNpc and a 49% loss in TH-positive terminals in the striatum, similar to other published findings [12], and lesioned mice showed elevated GFAP-labelled astrocytes in SN.

We counted TH-positive neurons in SNpc in 3D using an optical fractionator, and the numbers found in normal animals and in lesioned animals matched well with previous studies [19,24,39]. Interestingly, we also quantified SNpc TH-positive neurons from whole-slide scans with simple 2D counting. We did not use a correction, for example, the Abercombie correction [55], as this is appropriate if successive samples are counted because it is used to compensate for lost caps, and our sections were a 1:6 stereological series. The number of SNpc TH-positive neurons was lower, and the relative difference between groups declined. Thus, we note that 2D-based counts are less sensitive than traditional 3D optical fractionator-based counts [40].

Intrastriatal rotenone caused robust motor impairments in hindlimb stepping and motor coordination and balance on the accelerating rotarod paradigm. These behavioural tests are legitimate and sensitive tests of striatal integrity, and performance in hindlimb stepping is improved with levodopa [29,30]. However, we note that the rotarod paradigm showed large variability and required large group sizes. We detected minor changes in gait, which is reminiscent of the relative lack of sensitivity of this task when SNpc TH-positive neuronal loss is less than 80–90% [43,44]. Forelimb grip strength was unaffected in the rotenone-treated group. Interestingly, all-four-paw grip strength tended to be lower in the rotenone-treated group but was inconsistently statistically significant. This again indicates a lack of sensitivity of this test. PD patients show a decline in hand-grip strength with increasing UPDRS score or Hoehn and Yahr scale, which is independent of years since diagnosis [56,57], but again, almost 70% loss in TH-positive neurons in SNpc is noted in human tissue from PD patients (see above) [54]. No change was noted in spontaneous activity, again showing this task’s relative lack of sensitivity to 50% loss in TH-positive SNpc neurons. Thus, our rotenone-treated mice were Parkinsonian but may not have been as impaired as patients diagnosed with PD because their loss in dopamine neurons was reduced compared with typical PD patients. This was a great advantage, as it enabled the study of non-motor signs at early disease progression (based upon the loss of dopamine neurons alone).

A well-known non-motor prodromal symptom of PD is GI dysfunction [3,4,46], with a prevalence of 70–80% and characterised by bloating and delayed gastric emptying [58]. We examined GI motility using carmine red, a non-absorbable dye that is well-validated for whole gastrointestinal transit time in humans [48] and rodents [32,49]. We never observed delayed expulsion of carmine-red-coloured faeces in our rotenone-treated mice. Further, we observed no change in GFAP expression in the colon or duodenum, which is a marker of enteric glial cells that is elevated in PD [50]. Importantly, repeated testing further increased the robustness of our data, and, indeed, a lack of repeated testing has been identified as an issue in PD preclinical research [25]. As stated above, we define our model as early parkinsonism because we observed 50% loss in TH-positive neurons in SNpc (patients typically show 70% loss), and we observed consistent, robust, and specific motor deficits on the accelerating rotarod and in the hindlimb stepping task, only. Moreover, because expected peripheral signs (reduced GI motility) were absent, mice were Parkinsonian but did not model PD.

In this traditional model of PD, our primary goal was to examine sensations of heat, cold, and touch as disorders of the skin are being increasingly recognised in PD [5]. The skin of PD patients shows aggregated alpha-synuclein [7], and we have shown extensive alterations in the skin transcriptome of PD patients [59]. A recent meta-analysis revealed, in particular, increased sensitivity to cold pain in PD patients [52]. Incorporating sensory assessments alongside traditional motor assessments can provide a more complete picture of the disease, especially as counts of intraepidermal nerve fibres decline as PD progresses [60]. Interestingly, sensory neuronal impairment may explain, in part, some of the motor deficits in PD [61]. Additionally, sensory deficits are important prior to disease: skin complaints rise in the years prior to the diagnosis of PD [5] and peripheral neuropathy is observed in PD patients at diagnosis [62]. However, we did not observe any behavioural changes in heat, cold, or touch sensitivity in our mice. PD patients are much less likely to show changes in sensation in the area of the face and are more likely to show changes in upper or lower limbs [52]; thus, we focused on sensation in distal limbs. Again, we used statistically well-powered and well-validated behavioural tests, and we tested mice repeatedly. Thus, our data are clear indications of the failure of central DA loss alone to replicate PD, although it does mirror Parkinsonism (motor phenotypes). These data are reminiscent of data from primates, showing that MPTP, which primarily kills DA neurons in the brain, fails to cause extensive changes in gut motility or may even increase it [63]. Additionally, previous sensory testing revealed increased sensitivity to heat or pin-point pressure in rats lesioned with 6-OHDA [64,65], another traditional method of inducing dopaminergic cell loss in the SNpc. However, PD patients show increased sensitivity to cold pain most consistently [52].

Our data show that it is essential to take a comprehensive approach that considers the complexity of PD rather than dopaminergic neuronal loss. A recent review showed that the most typical models used to test potential disease-modifying therapeutics were toxic models (MPTP or 6-OHDA) [25]. Moreover, sample size calculations were “uniformly absent” from the papers they reviewed. A lack of sample size calculations also occurs in preclinical Alzheimer’s disease research [66] and in preclinical research on traumatic brain injury [67]; however, for example, we recently published sample sizes and expected power for many outcome measures for preclinical Alzheimer’s disease research [27], and we present further calculations in this paper. Certainly, MPTP and 6-OHDA models will develop central dopaminergic cell death and associated motor disorders, but our data reiterate that this is not sufficient to model the disease. Moreover, our data suggest that non-motor signs may not respond to levodopa, which has been shown to be the case in human PD patients [4], and indeed, high-dose levodopa may even contribute to peripheral neuropathy in PD [68,69].

In conclusion, our data show the importance of repeated testing in preclinical studies to reduce variability and group sizes for several behavioural outcome measures. Our behavioural testing used well-powered and well-validated tests that were suited to rodents but that have high translational relevance to humans [67,70]. Critically, and most importantly, our data show no peripheral behavioural effects following central administration of rotenone. Thus, central modelling does not reproduce expected peripheral signs of PD (gut motility) and also fails to induce sensory changes, which are increasingly recognised in PD but are little understood. Our data have important implications for current preclinical therapeutics testing in PD, which relies heavily on toxic central-based models; further, our data suggest that more comprehensive models are required to address peripheral signs in PD, which are a priority for development.

## Figures and Tables

**Table 1:** Group sizes required to detect a 50% or 30% difference from control, based upon single-timepoint testing or repeated testing.

Behavioral Outcome Measure	Group Size Required to See a 50% Change from Control α = 0.05 80% Power			Group Size Required to See a 30% Change from Control α = 0.05 80% Power		
	**Single-Timepoint Testing**		**Repeated Testing**	**Single-Timepoint Testing**		**Repeated Testing**
	**ClinCalc**	**G-Power**	**Glimmpse**	**ClinCalc**	**G-Power**	**Glimmpse**
Open field distance	6	7	10 ^1^	15	17	24 ^1^
Grip strength (all four paws)	0 ^2^	2	3 ^1^	1	3	4 ^1^
Grip strength (fore paws)	5	6	3 ^1^	13	14	5 ^1^
Hindlimb stepping	2	4	9 ^1^	5	7	23 ^1^
Rotarod	6	8	23 ^3^	18	19	62 ^3^
Tail flick	6	7	3 ^1^	16	17	4 ^1^
Hargreaves	3	5	9 ^1^	9	11	23 ^1^
von Frey	14	16	9 ^1^	39	41	23 ^1^
Cold plantar	6	7	9 ^4^	16	17	21 ^4^
Hot and and cold preference	11	13	7 ^5^	30	32	16 ^5^
Gut motility	4	6	7 ^1^	11	12	18 ^1^

^1^ Four timepoints including baseline. ^2^ Likely anomaly due to very high power. ^3^ Nine timepoints including baseline. ^4^ Based upon two timepoints, data per hind paw combined for analysis. ^5^ Based upon two timepoints, percent time spent at 32 °C used for analysis.

## Data Availability

All relevant data are within the paper and its Supplementary Materials.

## Supplementary Materials

The following supporting information can be downloaded at: https://www.mdpi.com/article/10.3390/cells13100799/s1, S1 file, which contains a graphical representation of 2D counts of SNpc TH-positive neurons, obtained via SlideViewer from whole-slide scans. S2 file contains all the data for this paper.

## Abbreviations

6-OHDA	6-hydroxydopamine
GFAP	glial fibrillary acidic protein
GI	gastrointestinal
LAMP1	Lysosomal-associated membrane protein
MPTP	1-methyl-4-phenyl-1,2,3,6-tetrahydropyridine
PD	Parkinson’s disease
PGP9.5	protein gene product 9.5 also ubiquitin carboxyl-terminal hydrolase-1
PN	peripheral neuropathy
ROI	region of interest
SNpc	*Substantia nigra pars compacta*
SNpr	*Substantia nigra pars reticulata*
