# Supplementary material for: No Evidence of Sensory Neuropathy in a Traditional Mouse Model of Idiopathic Parkinson’s Disease
Source: Cells. 2024 May 8;13(10):799. doi: 10.3390/cells13100799 (PMC11120514; doi:10.3390/cells13100799)
Supplement: Supplementary file 1 [file cells-13-00799-s001.zip › S1 file.pdf]

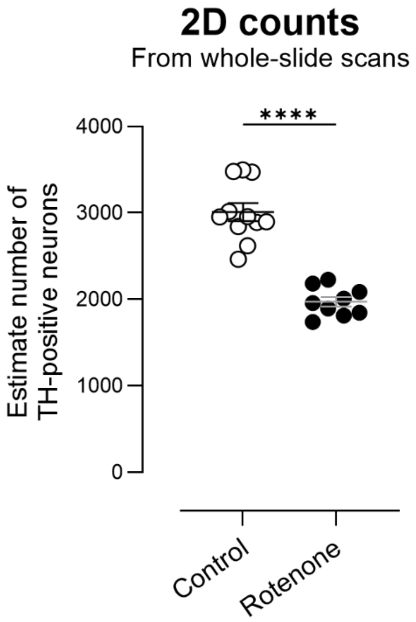

S1 file. Counts of TH-positive neurons in SNpc from whole-slide scans of a stereological series (1:6) through the entire SNpc. ****$p<0.0001$ (Mann Whitney test). Symbols are of individual mice, and horizontal bars show mean ± SEM.
